# Progression of fibrosing interstitial lung disease

**DOI:** 10.1186/s12931-020-1296-3

**Published:** 2020-01-29

**Authors:** Alyson W. Wong, Christopher J. Ryerson, Sabina A. Guler

**Affiliations:** 1 Department of Medicine, University of British Columbia, Vancouver, Canada; 2 Centre for Heart Lung Innovation, University of British Columbia, Vancouver, Canada; 3 Department of Pulmonary Medicine, Inselspital, Bern University Hospital, University of Bern, Freiburgstrasse 18, 3010 Bern, Switzerland

**Keywords:** Interstitial lung disease, Pulmonary fibrosis, Progression, Outcomes, Disease classification, Pulmonary function tests

## Abstract

Fibrotic interstitial lung diseases (ILDs) are often challenging to diagnose and classify, but an accurate diagnosis has significant implications for both treatment and prognosis. A subset of patients with fibrotic ILD experience progressive deterioration in lung function, physical performance, and quality of life. Several risk factors for ILD progression have been reported, such as male sex, older age, lower baseline pulmonary function, and a radiological or pathological pattern of usual interstitial pneumonia. Morphological similarities, common underlying pathobiologic mechanisms, and the consistently progressive worsening of these patients support the concept of a progressive fibrosing (PF)-ILD phenotype that can be applied to a variety of ILD subtypes. The conventional approach has been to use antifibrotic medications in patients with idiopathic pulmonary fibrosis (IPF) and immunosuppressive medications in patients with other fibrotic ILD subtypes; however, recent clinical trials have suggested a favourable treatment response to antifibrotic therapy in a wider variety of fibrotic ILDs. This review summarizes the literature on the evaluation and management of patients with PF-ILD, and discusses questions relevant to applying recent clinicial trial findings to real-world practice.

## Introduction

Interstitial lung disease (ILD) is a heterogeneous group of diseases characterized by inflammation and fibrosis of the lung parenchyma [1]. An important subset of patients with fibrotic ILD experience a decline in lung function with progressive symptoms, poor response to treatment, and reduced quality of life. Idiopathic pulmonary fibrosis (IPF) is the most common, severe, and progressive subtype of idiopathic interstitial pneumonia [2]; however, other ILD subtypes also have a progressive fibrosing phenotype. These include connective tissue disease-associated ILD (CTD-ILD) [3–5], fibrotic hypersensitivity pneumonitis (HP), unclassifiable ILD, idiopathic non-specific interstitial pneumonia (NSIP), and rarely sarcoidosis, organizing pneumonia, and ILD associated with occupational exposures.

The current classification of ILD focuses on identifying the underlying etiology since this frequently impacts both management decisions and prognostication [1, 6–9]. The distinction between IPF and non-IPF ILDs is particularly important given the worse prognosis in IPF compared to other fibrosing ILDs [9], and the different approaches to pharmacotherapy. IPF is primarily a fibrotic ILD, while fibrosis in non-IPF ILDs is often preceded or associated with inflammation. These inflammatory pathways can lead to activation of fibroblasts and their differentiation into myofibroblasts, which produce extracellular matrix that perpetuates remodelling of healthy lung tissue to pulmonary fibrosis [10]. Despite important differences, distinct ILD subtypes often have overlapping morphological features and common pathological mechanisms, leading to the concept of a progressive fibrosing phenotype that can be applied to a variety of fibrotic ILDs [1]. Recent evidence has supported this concept by suggesting some shared biological mechanisms and greater overlap in treatment options compared to historical approaches [11–15].

In this review, we summarize the current literature on the disease behaviour, progression of fibrosing ILD other than IPF, and approaches to management of patients with fibrosing ILD and a progressive phenotype. We discuss the clinical utility of ILD classification according to disease behavior, potential definitions of progressive fibrosing ILD (PF-ILD), and challenges for clinical decision making in the context of emerging treatment possibilities in patients with PF-ILD.

## Definition of PF-ILD

Patients with PF-ILD typically have self-perpetuating fibrosis characterized by worsening lung function, dyspnea, physical performance, and quality of life, as well as a poor response to therapy and early mortality [16]. Approximately 20–30% of patients with ILD are estimated to have PF-ILD [3–5]; however, there is no standardized definition of PF-ILD that clinicians and researchers have agreed upon. Several criteria have been used to define progression in patients with IPF, with most of these based on an absolute or relative decline in forced vital capacity (FVC) and diffusing capacity of the lung for carbon monoxide (DLCO) of ≥5–10% or ≥ 10–15% respectively, a decline in 6-min walk distance (6MWD) > 50 m, or worsening dyspnea and quality of life scores [8, 17–20]. In patients with systemic sclerosis (SSc)-associated ILD (SSc-ILD), an absolute 1-year decline in FVC ≥ 10% or 5–9% plus a decline in DLCO ≥15% are strong predictors of mortality [21]. In RA-ILD, an absolute FVC decline of ≥10% has been used as evidence of progression based on the increased mortality in patients meeting this threshold [22].

**Fig. 1:**
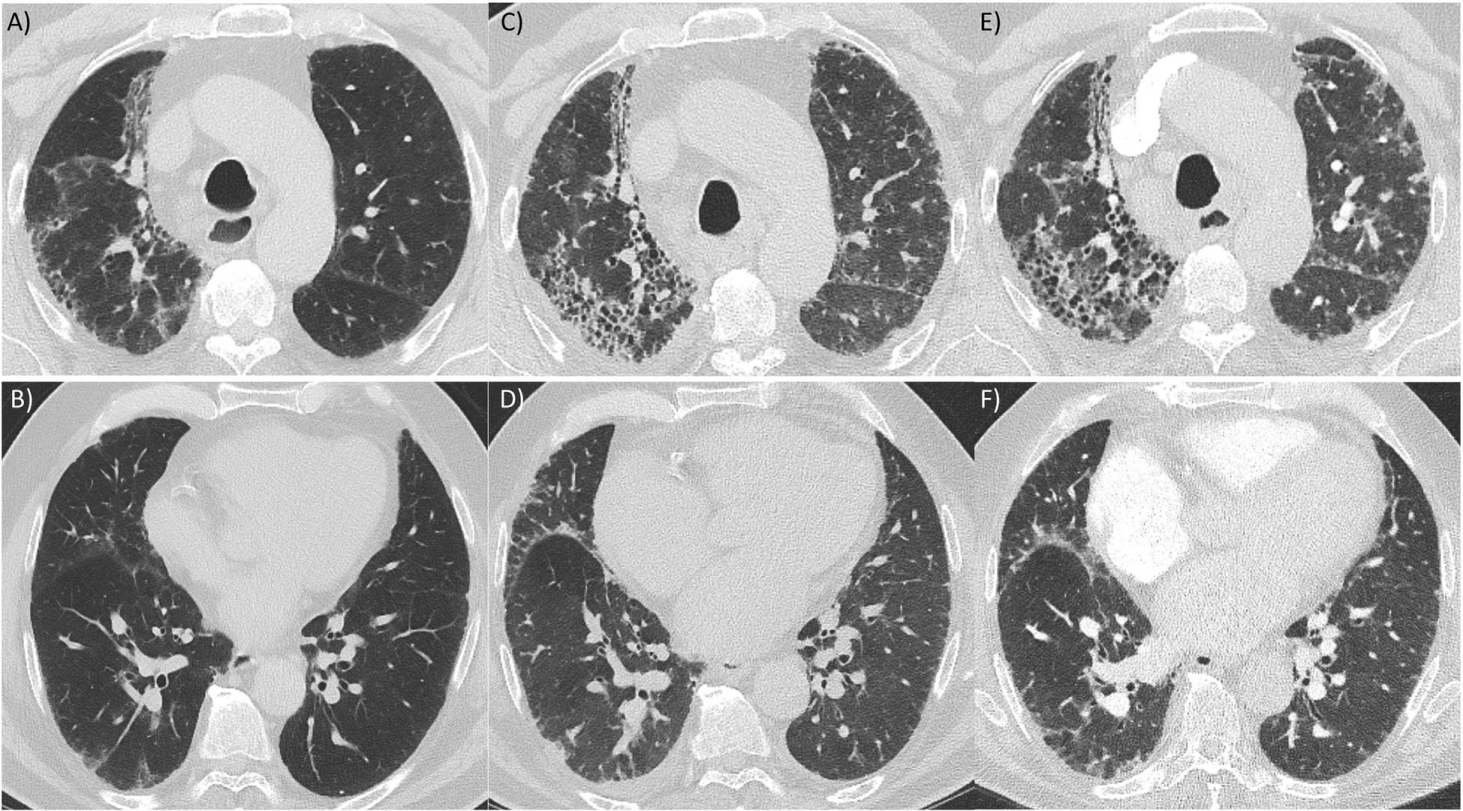
Computed tomography imaging of the chest in a patient with progressive unclassifiable interstitial lung disease. Serial apical (**a**, **c**, **e**) and basal (**b, d, f**) axial images at baseline (**a, b**); at 36 months (**c, d**); and at 42 months (**e, f**). Images show upper lobe predominant pulmonary fibrosis with progressive reticulation, traction bronchiectasis, and honeycombing

Despite these associations in large cohort studies, FVC and DLCO trajectories can be unpredictable and are less reliable among individual patients given important intra- and interpatient variability [4]. As a result, physicians typically use a combination of patient-reported symptoms, trends in pulmonary function tests, and the evolution of fibrosis on imaging (Fig. 1) to decide if there is clinically relevant ILD progression. Recently, several clinical trials have included patients with a progressive fibrosing phenotype, with the eligibility criteria for these studies helping to further guide a standardized definition of PF-ILD (Table 1) [14, 15, 25].

**Table 1:** Disease severity and definition of progression used in eligibility criteria for selected recently completed and ongoing clinical trials evaluating PF-ILD

Clinical trial	Disease severity		Minimum definition of progression			
	Pulmonary function	HRCT	Time frame	Pulmonary function	Symptoms	HRCT
Pirfenidone in unclassifiable ILD [15]	FVC ≥ 45% DLCO ≥30% 6MWD ≥ 150 m	Fibrosis affecting > 10% of lung volume	6 months	FVC > 5% decline (absolute)	Worsening symptoms	
Pirfenidone in progressive non-IPF ILD (RELIEF) [23]	FVC 40–90% DLCO 25–75% 6MWD ≥ 150 m		12 months^a^	FVC ≥ 5% decline (absolute)		
Nintedanib in non-IPF PF-ILD (INBUILD) [14]	FVC ≥ 45% DLCO 30–80%	Fibrosis affecting > 10% of lung volume	24 months	FVC ≥ 10% decline (relative)		
				At least two of:		
				FVC 5–10% decline (relative)	Worsening symptoms	Increasing extent of fibrosis
Pirfenidone in Patients With RA-ILD (TRAIL1) [24]	FVC ≥ 40% DLCO ≥30%	Fibrosis affecting > 10% of lung volume	12 months	FVC ≥ 10% decline (relative) or FVC 5–10% decline (relative) and DLCO ≥15% decline (relative)		

*Abbreviations*: *DLCO* diffusing capacity of the lungs for carbon monoxide, *FVC* forced vital capacity, *HRCT* high-resolution computed tomography, *ILD* interstitial lung disease, *IPF* idiopathic pulmonary fibrosis, *PF-ILD* progressive fibrosing ILD, *RA* rheumatoid arthritis, *6MWD* 6-min walk distance

^a^≥3 pulmonary function tests within 6–24 months, extrapolated to 12 months

## Risk factors for progression

Retrospective studies have identified risk factors that increase the likelihood of progression and mortality in PF-ILD including male sex, older age, lower FVC and DLCO at baseline, and certain morphological features [3, 6, 9, 21]. Specifically, a radiological and/or histological pattern of usual intersitial pneumonia (UIP) has been associated with increased mortality with poor prognosis also seen in patients with specific radiological features of UIP such as honeycombing and traction bronchiectasis [26–28]. The prognostic significance of a UIP pattern is seen across ILD subtypes, indicating that morphological pattern may be more prognostically important compared to the specific etiology of the disease [9, 29, 30].

An acute exacerbation is the most extreme type of ILD progression, characterized by a sudden and usually severe respiratory deterioration with new bilateral opacities on high-resolution computed tomography (HRCT) [31]. Risk factors for acute exacerbations in patients with IPF include severe dyspnea and low quality of life, higher body mass index, poor oxygenation, and low baseline FVC and DLCO. Patients with a recent relative decline in FVC ≥10% or DLCO ≥15% (hence PF-ILD by some definitions) are also more likely to experience an acute exacerbation [32–34]. Collectively, these findings suggest that patients with PF-ILD are at high risk for acute exacerbations. Recent studies have demonstrated the promise of specific blood biomarkers in predicting the risk of progression, acute exacerbations and mortality in patients with IPF and other PF-ILD, but additional prospective studies are needed to validate these findings and confirm their clinical utility [35, 36].

Similar to progression, mortality is challenging to predict for individual patients, in part related to the unpredictable occurrence of acute exacerbations and the high mortality from these events. The prognosis for patients with progressive ILD is worse than for stable and reversible ILD, although there is substantial variability across ILD subtypes even in patients with recent progression [37].

Most notably, patients with CTD-ILD and fibrotic HP appear to have a better prognosis than patients with IPF [9]. Additional data are needed to test whether this assumption of a worse prognosis in IPF remains valid when comparing to non-IPF patients who also have a progressive fibrotic phenotype.

## Management

### Non-pharmacologic treatment

The majority of non-pharmacological management strategies apply to all patients with fibrotic ILD, regardless of progression or the underlying etiology. Smoking cessation, antigen avoidance, protection from occupational exposures, and cessation of medications that can potentially cause ILD are all important. Pneumococcal vaccination and annual influenza vaccination are also appropriate in almost all patients with fibrotic ILD. With frequent loss of physical function and muscle mass in patients with severe and progressive ILD [38], pulmonary rehabilitation potentially relieves symptoms and improves exercise capacity [39]. Similarly, selected patients can benefit from long-term oxygen therapy [40]. Comorbidities and overall frailty can further reduce quality of life and survival, and patients are consequently likely to benefit from screening and management of important comorbidities [41, 42]. Early evaluation for lung transplant should be considered for patients with PF-ILD who are potentially eligible, while symptom-based management approaches including pallative care should take priority in patients with severe ILD who are not candidates for lung transplantation [43].

### Pharmacologic treatment

Patients with PF-ILD have generally been treated with either antifibrotic therapy for IPF or immunosuppressive therapy for non-IPF ILD. The distinction between IPF and non-IPF ILD is particularly important because immunosuppressive therapy is harmful for patients with IPF [8], but potentially beneficial for patients with non-IPF ILD [44, 45]. For this reason, increasing diagnostic certainty within a multidisciplinary team discussion can help clinicians make more informed treatment recommendations, and this process can also inform management even in patients who are left without a confident diagnosis [46, 47].

#### Antifibrotic therapy

Pirfenidone and nintedanib are antifibrotic medications indicated for the treatment of IPF based on high-quality randomized placebo-controlled trials [18, 19, 48, 49]. Both medications attenuate migration, differentiation, and activation of fibroblasts, which are key cells in the development and progression of pulmonary fibrosis [50]. Preclinical studies suggest an antifibrotic effect in pulmonary fibrosis caused by a variety of different triggers. Both agents also have anti-inflammatory properties [51, 52]. The clinical trials that led to the approval of nintedanib and pirfenidone in IPF demonstrated approximately 50% reduction in the rate of FVC decline in the treated group compared to placebo, with potential reduction in the risk of exacerbation and death [18, 19, 49]. Subgroup analyses from the pirfenidone and nintedanib clinical trials showed consistent treatment effects regardless of demographics and disease severity [53, 54]; and post-hoc analyses from nintedanib trials have further suggested potential efficacy in a broader population of patients with a multidisciplinary diagnosis of IPF who did not meet IPF diagnostic criteria from recent clinical practice guidelines [55].

The SENSCIS trial was the first phase III trial to investigate the efficacy and safety of nintedanib in patients with a non-IPF ILD [56], and based on this trial the U.S. Food and Drug Administration recently approved nintedanib for the treatment of SSc-ILD. Patients within 7 years of their first non-Raynaud SSc manifestation with at least 10% fibrosis on HRCT were included in the study. A stable background therapy with mycophenolate mofetil (MMF) was permitted, whereas patients with severe pulmonary hypertension or digital ulcers were excluded. Analysis of the primary outcome showed approximately 50% reduction in the rate of FVC decline in the treated group compared to placebo, but patients had overall less rapid progression compared to the IPF clinical trial populations [56]. There was no observed impact of treatment on Rodnan skin score or health-related quality of life. Tolerability of nintedanib was acceptable with a similar adverse effect profile compared to IPF patients. Diarrhea was the most frequent adverse effect, experienced by 76% of patients in the nintedanib and 32% of patients in the placebo group. The subgroup of patients receiving background MMF had less relative benefit from nintedanib compared to placebo (mean between-group difference 26 mL/year in patients on MMF versus 55 mL/year in patient without MMF).

An open-label phase 2 study showed acceptable adverse events profile of pirfenidone in patients with SSc-ILD [57]. Tolerability of pirfenidone was similar to patients with IPF, with a slightly better tolerability when pirfenidone was titrated over 4 weeks instead of 2 weeks.

In addition to SSc-ILD, recently published clinical studies have investigated the effect of nintedanib and pirfenidone in patients with unclassifiable and other non-IPF PF-ILDs, with similar study designs compared to the previous IPF trials (Table 2). The INBUILD trial showed that annual FVC decline in PF-ILD patients receiving nintedanib was reduced by 57% (107 ml) compared to placebo, with more than 60% reduction in FVC decline (128 ml) in patients who had a radiologic UIP-like pattern [14]. Patients in the INBUILD study were not permitted use of immunosuppressive medications, possibly suggesting a study population with less abundant inflammatory features. Several studies of pirfenidone have enrolled patients with a variety of non-IPF PF-ILD subtypes who have worsened despite therapy, including a study in unclassifiable ILD [15], and another study published only in abstract form that included patients with CTD-ILD, fibrotic idiopathic NSIP, fibrotic HP, and ILD associated with asbestosis [23]. In a recent phase 2 study evaluating pirfenidone in unclassifiable ILD, the safety profile of pirfenidone was reassuring with promising, but inconsistent efficacy signals that depended on both the endpoint and the method of statistical analysis. Specifically, FVC measured by daily home spirometry was chosen as the primary endpoint in this trial; however, these measurements were highly variable and prohibited performance of the planned statistical analysis. More reassuringly, results from spirometry performed at the study sites suggested a slower rate of FVC decline in patients on pirfenidone compared to placebo [15]. There are several ongoing studies that will further clarify the roles for nintedanib and pirfenidone in PF-ILD (Table 3). Regulatory bodies will soon make decisions on whether antifibrotic medications will receive indications for use in non-IPF ILDs.

**Table 2:** Major randomized controlled trials investigating antifibrotic or immunosuppressive treatments in patients with non-IPF ILD

Study	Patient population (sample size)	Treatment^a^	1^o^ endpoint	Selected 2^o^ endpoints
Antifibrotic medication				
SENSCIS (Phase 3) [56] NCT02597933	SSc-ILD (576)	Nintedanib	Nintedanib reduced the rate of FVC decline over *52 weeks* (mean between-group difference 41 mL; 95%CI 2.9-79 mL, *p* = 0.04), with smaller absolute benefit from nintedanib compared to IPF clinical trials.	No difference in mRSS and QOL.
INBUILD (Phase 3) [14] NCT02999178	PF-ILD (663): Fibrotic HP, CTD-ILD, iNSIP, unclassifiable ILD	Nintedanib	Nintedanib reduced the rate of FVC decline over *52 weeks* (mean between-group difference 107 mL; 95%CI 65-149 mL, *p* < 0.001), with similar absolute benefit from nintedanib compared to IPF clinical trials.	No difference in QOL and survival.
LOTUSS (Phase 2/Open-label) [57] NCT01933334	SSc-ILD (63)	Pirfenidone *(stable background therapy with MMF allowed)*	An adverse event occurred in 97% of patients over *16 weeks*. Patients in the 4 week titration group had improved tolerability compared to patients in the 2 week titration group. Tolerability was similar among patients on both MMF and pirfenidone compared to patients on pirfenidone only.	No difference in FVC, DLCO, patient-reported outcomes or mRSS between the titration groups.
RELIEF (Phase 2) [23] DRKS00009822	PF-ILD (127): CTD-ILD, fibrotic iNSIP, fibrotic HP, asbestosis	Pirfenidone *(added to conventional therapy)*	Pirfenidone reduced the rate of FVC decline over *48 weeks* when statistical analyses used imputed data.	No difference in DLCO, 6MWD, QOL, and safety profile.
Pirfenidone in unclassifiable ILD (Phase 2) [15] NCT03099187	Unclassifiable PF-ILD (253)	Pirfenidone *(stable background therapy with MMF allowed)*	The prespecified analysis could not be performed due to high variability for individual daily home spirometry readings.	Pirfenidone reduced the rate of FVC decline over *24 weeks* using site spirometry (mean between-group difference 95.3 mL, 95%CI 36-155 mL, *p* = 0.002). No differences in DLCO, 6MWD, and safety.
Immunosuppressive medication				
SLS (Phase 3) [45] NCT00004563	SSc-ILD (158)	CYC p.o.	CYC reduced the rate of FVC decline over *52 weeks* (mean between-group difference 2.53, 95%CI 0.28–4.79%, *p* < 0.03) after adjustment for baseline FVC.	More adverse events in the CYC group.
SLS II (Phase 3) [58] NCT00883129	SSc-ILD (126)	MMF for 2 years versus CYC p.o. for 1 year followed by placebo for 1 year	No between-group difference in FVC over *24 months* with a greater proportion of patients withdrawing from CYC than from MMF.	No between-group difference in mRSS, change in HRCT lung fibrosis scores, or dyspnea score.
FAST [59]	SSc-ILD (45)	6 months of CYC i.v. and prednisone followed by 6 months of AZA versus placebo	No between-group difference in FVC over *52 weeks* in this small, potentially underpowered study*.*	No between-group differences in HRCT fibrosis and dyspnea scores.
FOCUSSED (Phase 3) NCT02453256 *[presented in abstract form]*	SSc (210) including SSc-ILD (132)	Tocilizumab	No statistically significant difference in mRSS over *48 weeks* (adjusted mean between-group difference − 1.73, 95%CI −3.78-0.32, *p* = 0.098).	Tocilizumab reduced the rate of FVC decline over *48 weeks* on post-hoc analysis in the subgroup of patients with SSc-ILD (mean between-group difference 6.4, 95%CI 3.3–9.4%)

*Abbreviations*: *AZA* azathioprine, *CI* confidence interval, *CTD* connective tissue disease, *CYC* cyclophosphamide, *DLCO* diffusing capacity of the lungs for carbon monoxide, *FVC* forced vital capacity, *HP* hypersensitivity pneumonitis, *HRCT* high-resolution computed tomography, *ILD* interstitial lung disease, *iNSIP* idiopathic nonspecific interstitial pneumonia, *IPF* idiopathic pulmonary fibrosis, *i.v.* intravenous, *MMF* mycophenolate mofetil, *mRSS* modified Rodnan skin score, *p.o.* per os, *QOL* quality of life, *SSc* systemic sclerosis, *6MWD* 6-min walk distance

^a^compared to placebo unless otherwise stated

**Table 3:** Ongoing randomized controlled trials investigating antifibrotic and immunosuppressive treatments in non-IPF ILD

Study	Patient population (sample size)	Treatment^a^	1^o^ endpoint	Selected 2^o^ endpoints	Expected completion date
Antifibrotic medications					
SLS III (Phase 2) NCT03221257	SSc-ILD (150)	Pirfenidone *(added to MMF)*	Change in FVC over *18 months*	Change in DLCO, mRSS, QOL, Dyspnea	December 2021
Pirfenidone in SSc-ILD (Phase 3) NCT03856853	SSc-ILD (144)	Pirfenidone	Change in FVC over *52 weeks*		February 2021
TRAIL1 (Phase 2) NCT02808871	RA-ILD (270)	Pirfenidone	Incidence of composite endpoint of FVC decline ≥10% or death over *52 weeks*	Frequency of progressive fibrosis *(FVC decline ≥ 10%, or 5–10% and DLCO decline ≥ 15%)*	November 2021
PirFS NCT03260556	PF-sarcoidosis (60)	Pirfenidone	Time to clinical worsening over *24 months*	Change in FVC and composite physiologic index	December 2019
Pirfenidone Fibrotic HP NCT02958917	Fibrotic HP (40)	Pirfenidone	Change in FVC over *52 weeks*	Progression-free survival^b^	December 2019
Pirfenidone in DM-ILD (Phase 3) NCT03857854	DM-ILD (152)	Pirfenidone	Change in FVC over *52 weeks*		February 2021
Immunosuppressive medications					
RECITAL (Phase 2–3) NCT01862926	CTD-ILD (116)	Rituximab versus CYC	Change in FVC over *48 weeks*	Adverse events, change in DLCO and QOL	November 2020
Bortezomib and MMF in SSc-ILD (Phase 2) NCT02370693	SSc-ILD (30)	Bortezomib *(added to standard of care immunosuppression)*	Safety over *24 weeks*	Change in FVC, mRSS, QOL	June 2020
EvER-ILD (Phase 3) NCT02990286	Idiopathic or CTD-associated NSIP (non-responders to first-line immunosuppression) (122)	Rituximab *(added to standard of care immunosuppression)*	Change in FVC over *6 months*	Change in 6MWD, DLCO, dyspnea, cough	June 2020
ATtackMy-ILD (Phase 2) NCT03215927	Myositis associated ILD (20)	Abatacept (added to standard of care immunosuppression)	Change in FVC over *24 weeks*	Progression-free survival^c^ and change in dyspnea	December 2020

*Abbreviations*: *CTD* connective tissue disease, *CYC* cyclophosphamide, *DLCO* diffusing capacity of the lungs for carbon monoxide, *DM* dermatomyositis, *FVC* forced vital capacity, *HP* hypersensitivity pneumonitis, *HRCT* high-resolution computed tomography, *ILD* interstitial lung disease, *MMF* mycophenolate mofetil, *mRSS* modified Rodnan skin score, *NSIP* idiopathic nonspecific interstitial pneumonia, *QOL* quality of life; SSc, systemic sclerosis, *6MWD* 6-min walk distance

^a^compared to placebo unless otherwise stated

^b^time to FVC %-predicted decline ≥5%, or 6MWD decline ≥50 m, or progression of fibrosis on HRCT, or acute exacerbation

^c^time to FVC%-predicted decline ≥10%, or ≥ 5% and DLCO %-predicted decline ≥15%, or death or lung transplantation

#### Immunosuppressive therapies

Immunosuppressive medications are not appropriate in the chronic management of IPF given the increased risk of mortality and other adverse consequences, without any clear benefit [8]. Based on these findings, immunosuppressive medications are used with great caution in patients with an IPF-like phenotype. Conversely, these medications are generally considered appropriate in many patients with CTD-ILD or fibrotic HP, and in some patients with unclassifiable ILD (Table 2) [44].

##### Connective tissue disease-associated ILD

Most evidence for the use of immunosuppressive medications in CTD-ILD is extrapolated from the Scleroderma Lung Study [45]. This study showed that patients receiving cyclophosphamide had a slower rate of FVC decline compared to placebo, with a mean absolute difference of 2.5% between groups after 1 year of therapy. However, concerns of toxicity, low tolerability, and loss of effect after 18 months led to the Scleroderma Lung Study II which compared 2 years of MMF to 1 year of oral cycphosphamide [58]. Although MMF and cyclophosphamide had similar efficacy, MMF had less hematotoxicity and substantially fewer premature study withdrawals. MMF is consequently the preferred initial and maintenance immunosuppressive agent for most patients with SSc-ILD [60]. Besides cyclophosphamide and MMF, other frequently used medications in CTD-ILD include azathioprine, methotrexate, and rituximab.

Tocilizumab is a subcutaneously administered interleukin 6 receptor-α inhibitor that was recently studied in phase II and III trials in patients with early and progressive SSc. The studies did not meet their primary endpoint, which was the difference in change of the modified Rodnan skin score between groups [57, 61]. Recent results from exploratory and post-hoc analyses revealed that fewer SSc-ILD patients in the tocilizumab group had a decline in FVC at 48 weeks, with quantitative lung fibrosis scores also supporting potential benefit from tocilizumab in this subgroup.

##### Fibrotic hypersensitivity pneumonitis

Immunosuppressive medications are frequently used in patients with fibrotic HP despite the absence of prospective controlled trials. In patients with non-fibrotic HP, short courses of prednisone combined with exposure elimination may be sufficient to halt and even reverse the disease process. Management is more challenging in fibrotic HP, with many patients having a progressive fibrotic phenotype even with exposure elimination. Based on retrospective comparisons in patients with fibrotic HP, both MMF and azathioprine are well-tolerated, potentially reduce the need for prednisone, and may improve the trajectory of DLCO decline [44]. Despite the limited data, MMF and azathioprine are typically considered first-line options for patients with fibrotic HP who have had or are at risk of ongoing progression.

##### Unclassifiable ILD

Approximately 12% of patients with ILD cannot be classified after a diagnostic workup, and management is particularly challenging in this patient population [62]. Unclassifiable ILD is a diverse collection of patients who frequently have features resembling CTD-ILD or fibrotic HP. The concept of a working diagnosis has been suggested as a useful approach in deciding upon the most appropriate management of these patients [46]. Depending on the relative likelihoods of different diagnoses in a patient with unclassifiable ILD, either antifibrotic or immunosuppressive therapy may be warranted. The use of immunosuppressive therapies in this situation is based on very limited retrospective data suggesting potential benefit from cyclophosphamide in the subgroup of unclassifiable ILD patients who have autoimmune features [63, 64].

Additional clinical trials are ongoing that might provide further evidence on the efficacy and safety of immunosuppressive therapies in patients with non-IPF ILDs (Table 3).

## Discussion

The disease behaviour of various ILD subtypes has been studied in many observational cohorts and clinical trials, with many of these studies focusing on patients with a progressive fibrotic phenotype. The eligibility criteria of these studies have provided a starting point for the definition of PF-ILD [14, 15, 23, 24], with the clinical utility of this concept supported by the therapeutic benefits observed in some of these clinical trials [14, 15]. Despite these significant advances, there are many uncertainties that still remain in the classification and management of PF-ILD.

### Is the concept of PF-ILD clinically useful?

Establishing a confident ILD diagnosis is often challenging, with considerable heterogeneity in diagnoses assigned by different experienced multidisciplinary teams [47], and approximately 12% of patients remaining unclassifiable after a thorough evaluation including a surgical lung biopsy [62]. Although diagnostic certainty is often low in ILD, it is typically still feasible to characterize disease behaviour using symptoms or routine investigations such as pulmonary function tests and chest imaging.

There is therefore appeal to simply classifying patients with ILD based on previous or anticipated disease behaviour rather than needing to establish a confident diagnosis; however, there are also important disadvantages of this approach. First, this approach could detract from a thorough assessment for an underlying etiology, with the danger that crucial management steps such as antigen avoidance might be neglected, or that specific pharmacotherapy options may not be considered. Second, creation of such a large and heterogeneous cohort would complicate development and testing of targeted therapies that apply to specific subgroups. Finally, translation of clinical trial findings from a heterogenuous cohort to individual patients might be challenging, with limited ability to understand whether clinical trial results are being driven by a specific subset of patients.

Despite these concerns, recent clinical trials support the clinical utility of PF-ILD by demonstrating potential treatment implications of this designation [14, 15]. It is therefore likely that the concept of PF-ILD will become further incorporated into clinical practice; however, with the important caveat that disease behaviour should be complementary to the current approach to ILD classification, rather than replacing it.

### How should PF-ILD be defined?

There are no established diagnostic criteria available to determine disease progression or a definition of PF-ILD. Clinical trials have consistently defined PF-ILD based on worsening symptoms, pulmonary function, or imaging; however the specific combination of features and thresholds vary across studies (Table 1).

Decline in pulmonary function is a key criterion for PF-ILD in all studies, with a relative decline in FVC ≥10% being a common threshold for progression when used in isolation. Smaller changes in FVC (e.g. relative decline of 5–10%) are less specific [4, 65], and are typically complemented by worsening symptoms or imaging findings, which are often defined qualitatively. Most recent studies have not included DLCO in their definition of PF-ILD; however, previous studies and working group statements have proposed ≥15% decline in DLCO is clinically meaningful, with smaller changes often used in combination with other markers of ILD progression [20, 21].

Criteria for progression that are used in recent studies may not translate directly into a clinical setting. Notably, imaging studies are not feasible at all follow-up visits, and thus radiological progression will likely be a relatively infrequent criterion of disease progression in clinical practice. In addition, experienced clinicians frequently integrate these markers of progression in a more complex manner than what can feasibly be replicated in a clinical trial that prioritizes standardization. Finally, clinicians have many other markers of ILD progression to consider on an individual basis, including exercise capacity, oxygenation, and respiratory hospitalization. It is therefore likely that there will be some differences in how PF-ILD has been defined in clinical trials compared to how clinicians will define this subgroup in a real-world setting.

### How should clinicians choose between antifibrotic or immunosuppressive therapy in patients with non-IPF PF-ILD?

Antifibrotic and immunosuppressive medications are both currently off-label for patients with non-IPF PF-ILD; however, recent data have generated interest in using these medications in some of these patients pending the necessary regulatory approvals.

In the future, potential management options for patients with PF-ILD will likely include no specific pharmacological treatment, immunosuppressive medication, antifibrotic medication, or a combination of these treatments.

The option to forgo initiation of both immunosuppressive and antifibrotic therapy will usually apply to patients with significant comorbidities, those who are likely to tolerate potential adverse effects poorly, patients in a palliative setting, and those who decline pharmacotherapy for other reasons. The choice between antifibrotic and immunosuppressive medication in PF-ILD has not been answered by recent clinical trials, as none of these studies have directly compared these two options [14, 15]. In the absence of robust head-to-head data, it will likely be most appropriate to consider antifibrotic medication in patients with an IPF-like phenotype, particularly those with a UIP pattern on chest imaging or lung biopsy, as well as patients for whom immunosuppression might be associated with greater potential adverse effects. Using immunsuppressive therapy as a first-line option is likely to be most beneficial in patients with a more inflammatory phenotype, and particularly those with an organising pneumonia pattern on chest imaging and other features of active autoimmunity [63, 64]. There is a substantial “grey zone” of patients with fibrotic ILD who fall between these two extremes. Decisions in these patients will be more challenging, and should generally be supported by a multidisciplinary discussion as well as close communication with rheumatologists in patients with features of a CTD. In this situation, some clinicans use a brief trial (e.g., 1–2 months) of prednisone to identify potential candidates for long-term immunosuppression, prioritizing ongoing immunosuppression in patients who respond favourably to this shorter trial. The pragmatic assumption that short-term response to prednisone translates into long-term benefit of immunsuppressive medication needs to be established in controlled trials, and there is also the important downside that this approach is very likely harmful in patients with an underlying biology similar to IPF [8]. This approach should thus be used with caution. Lastly, the combination of antifibrotic and immunosuppressive medications is a potential option given that both therapies are targeting different biological pathways involved in the biology of non-IPF PF-ILD. Recent studies have shown that the combination of pirfenidone and MMF is safe in patients with unclassifiable ILD and similarly for nintedanib and MMF in SSc-ILD [15, 56].

Many questions remain to be answered in future studies of PF-ILD: Which antifibrotic and immunosuppressive therapies can be combined safely? What is the incremental benefit of these medications when added to an established background therapy? Should antifibrotics and immunosuppressants be combined upfront or sequentially only in the context of ongoing disease progression? How should response to these treatments be assessed? And how should the additional therapeutic burden be managed, particularly, in polymorbid and frail patients? Additional clinical trials are forthcoming that will provide further insight into these questions, as well as anticipated post-hoc analyses from recently published studies.

## Conclusion

The term PF-ILD identifies a subgroup of patients with ILD who often have a poor prognosis, with recent clinical trials suggesting potential benefit from antifibrotic medication in this population. These trials support the utility of subclassifying patients with various fibrotic ILD subtypes based on their anticipated disease behaviour; however, it is critical to remember that this approach is complementary to establishing a confident clinical diagnosis that also carries important management and prognostic implications. Although recent advances have suggested exciting new options for patients with fibrotic ILD, many questions remain and collaborative efforts are needed to address these issues.

## Data Availability

Not applicable.

## Abbreviations

6MWD: 6-min walk distance
AZA: Azathioprine
CI: Confidence interval
CTD: Connective tissue disease
CYC: Cyclophosphamide
DLCO: Diffusing capacity of the lungs for carbon monoxide
DM: Dermatomyositis
FVC: Forced vital capacity
HP: Hypersensitivity pneumonitis
HRCT: High-resolution computed tomography
i.v.: Intravenous
ILD: Interstitial lung disease
IPF: Idiopathic pulmonary fibrosis
MMF: Mycophenolate mofetil
mRSS: Modified Rodnan skin score
NSIP: Nonspecific interstitial pneumonia
p.o.: Per os
PF-ILD: Progressive fibrosing interstitial lung disease
QOL: Quality of life
RA: Rheumatoid arthritis
SSc: Systemic sclerosis
UIP: Usual interstitial pneumonia
